# Rupture point is associated with divergent hemodynamics in intracranial aneurysms

**DOI:** 10.3389/fneur.2024.1364105

**Published:** 2024-05-20

**Authors:** Aleš Hejčl, Jana Brunátová, Helena Švihlová, Jan Víteček, Andrea Vítečková Wünschová, Alena Sejkorová, Mária Hundža Stratilová, Tomáš Radovnický, Martin Sameš, Jaroslav Hron

**Affiliations:** ^1^Department of Neurosurgery, Masaryk Hospital, J. E. Purkyne University, Ústí nad Labem, Czechia; ^2^International Clinical Research Center, St. Anne’s University Hospital Brno, Brno, Czechia; ^3^Institute of Experimental Medicine of the Czech Academy of Sciences, Prague, Czechia; ^4^Faculty of Mathematics and Physics, Mathematical Institute, Charles University, Prague, Czechia; ^5^Faculty of Science and Engineering, Bernoulli Institute, University of Groningen, Groningen, Netherlands; ^6^Institute of Biophysics of the Czech Academy of Sciences, Brno, Czechia; ^7^Department of Biochemistry, Faculty of Medicine, Masaryk University Brno, Brno, Czechia; ^8^Department of Anatomy, Faculty of Medicine, Masaryk University Brno, Brno, Czechia

**Keywords:** intracranial aneurysm, computational fluid dynamics, rupture, particle image velocimetry (PIV), wall shear stress (WSS)

## Abstract

**Background:**

Understanding the risk factors leading to intracranial aneurysm (IA) rupture have still not been fully clarified. They are vital for proper medical guidance of patients harboring unruptured IAs. Clarifying the hemodynamics associated with the point of rupture could help could provide useful information about some of the risk factors. Thus far, few studies have studied this issue with often diverging conclusions.

**Methods:**

We identified a point of rupture in patients operated for an IAs during surgery, using a combination of preoperative computed tomography (CT) and computed tomography angiography (CTA). Hemodynamic parameters were calculated both for the aneurysm sac as a whole and the point of rupture. In two cases, the results of CFD were compared with those of the experiment using particle image velocimetry (PIV).

**Results:**

We were able to identify 6 aneurysms with a well-demarcated point of rupture. In four aneurysms, the rupture point was near the vortex with low wall shear stress (WSS) and high oscillatory shear index (OSI). In one case, the rupture point was in the flow jet with high WSS. In the last case, the rupture point was in the significant bleb and no specific hemodynamic parameters were found. The CFD results were verified in the PIV part of the study.

**Conclusion:**

Our study shows that different hemodynamic scenarios are associated with the site of IA rupture. The numerical simulations were confirmed by laboratory models. This study further supports the hypothesis that various pathological pathways may lead to aneurysm wall damage resulting in its rupture.

## 1 Introduction

In clinical practice there has been a substantial increase in the detection of unruptured intracranial aneurysms (IA) within the population due to growing use of non-invasive neuroimaging diagnostic methods (1). This creates a burden not only for the patients themselves, but for the clinicians as they lack precise indicators for optimal therapy, when consulting these patients. Computational fluid dynamics (CFD) has gained increasing interest in the clinical community, as it could potentially clarify the pathophysiology of IA development, growth, and rupture. Some hemodynamic parameters have already been integrated in newly developed scoring systems (2, 3). One of the first grading systems called rupture resemblance score (RRS) system was proposed recently by Meng et al. utilizing a combination of hemodynamic and morphological features of ruptured IAs (4).

Few studies have focused on local hemodynamics with respect to the site of rupture (5, 6). Modeling the local hemodynamic environment could reveal key information in understanding its role in aneurysm rupture. On the other hand, a low number of studies is available, which is most likely due to the fact that it is often complicated or even impossible to identify the precise point of rupture in an IAs. Proper identification of the point of rupture can be performed at several points—during surgical exploration of the IA, during clipping or during an endovascular diagnostic procedure, if periprocedural rupture occurs. Thus far, ruptured IAs have been associated with both low and high WSS. The relationship between WSS and aneurysmal rupture is complex and not fully understood (7–9). The relationship between the point of rupture and hemodynamics is even less clear (5).

Despite a growing body of literature on hemodynamics and IAs, it has little impact on the clinical decision-making regarding patients with unruptured IAs. This may be related to the complexity of the mathematical model as well as the uncertainty surrounding the role of individual hemodynamic parameters (10). Modeling the local hemodynamic environment could reveal key information on its role in aneurysm rupture and possibly bring our knowledge closer to clinical every-day reality (10).

## 2 Methods

### 2.1 Aneurysm selection/patient population

We included all patients operated for a ruptured intracranial aneurysm in whom the surgeon was able to identify the point of rupture on preoperative computed tomography (CT) angiography. Altogether, six aneurysms with a known rupture site were collected within 2 years. All patients s also needed to have a high-quality preoperative CT image available, and these acted as the bases for computational meshes. Their sizes varied from 8.83 to 12.60 mm, and they were located on the middle cerebral artery (5 cases) and right internal carotid artery bifurcation (1 case) as shown in the Table 1. Aneurysm size was calculated as the maximum distance within the aneurysm dome, the diameter of the neck was determined as the maximum distance in the neck plane, and the aspect ratio calculated as the ratio between the height (maximum perpendicular height of the aneurysm) and the diameter of the neck. In aneurysm F, the neck was not defined.

**Table 1:** Morphology of aneurysms and their location in the brain.

Aneurysm designation	Location	Size (mm)	Volume (mm^3^)	Surface area (mm^2^)	Neck diameter (mm)	Aspect ratio (-)
A	Right carotid (C5)	10.56	205	566	5.42	1.57
B	Left MCA	9.96	182	920	6.35	1.14
C	Left MCA	9.22	145	626	4.67	1.62
D	Left MCA	12.60	332	987	7.92	1.20
E	Right MCA	9.84	183	447	5.23	1.46
F	Right MCA	8.83	133	364	—	—

### 2.2 Image segmentation

CT angiography was performed at admission using 256-detector multislice CT, with a pixel resolution of 0.6 mm. CT acquisition was synchronized with the contrast bolus throughout a large cranio-caudal, or *z*-axis, field of view. All CT angiographies were performed after IA rupture and therefore the shape of the aneurysm may have not reflected the pre-rupture conditions. The data were then used for segmentation and 3D visualization of the intracranial blood vessels using ITK-SNAP (11). A 3D model of the aneurysm sac as well as the major inlet and outlet blood vessels was constructed.

### 2.3 Mesh generation

Voxel segmentations of patient-specific geometries of cerebral aneurysms obtained from the CT image slice were interactively constructed by the ITK-SNAP program. At least two medical doctors approved this first 3D reconstruction independently. After binary voxel segmentation, the surface meshes were constructed and smoothed using a combination of lowpass and Laplace smoothing filters as implemented in the iso2mesh package (12). The parent vessel was modeled as long as possible, at least six times longer than the inlet diameter of the vessel (13), to achieved fully developed flow. All inlet and outlet branches were extended to obtain cross-sections that were circular and almost perpendicular to the centerline of the vessel. All final surface meshes were approved by medical doctors.

Consequently, three tetrahedral meshes with various average edge lengths were constructed for all six aneurysm cases to test the mesh dependence. A mesh dependency test was performed for steady flow in each case with an input flux of 169 mL/min. Values of velocity magnitude, normal pressure, and wall shear stress were averaged over the aneurysm dome and compared among the three computations.

### 2.4 The model and boundary conditions

Navier–Stokes equations with constant dynamic viscosity *μ* = 3.5 mPa·s and constant blood density *ρ* = 1,050 kg·m^−3^ were considered to be the governing equations for blood flow. A fast and robust code was used for solving such equations by the finite element method, as described and evaluated by Chabiniok et al. (14). The program is implemented in Python using the FEniCS (15) and PETSc libraries (16). Time derivatives were approximated by the BDF scheme; the finite element method was used for spatial discretization of the velocity and pressure fields, in particular the MINI element was used (17).

**Figure 1:**
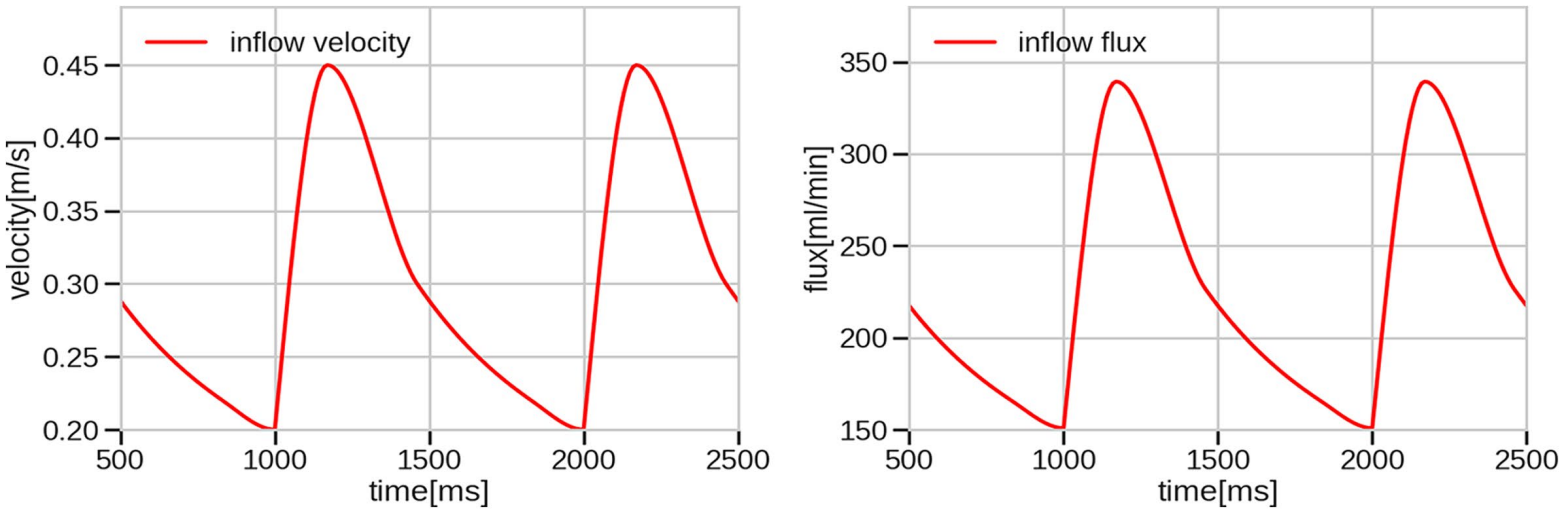
Inflow velocity waveform set for all cases and corresponding inflow flux waveform for radius = 2 mm.

The no-slip boundary condition was prescribed on the vessel wall and the zero surface-traction boundary condition on the outlets. The parabolic profile for the inlet velocity was prescribed on the circular inlet surface, with spatially averaged velocity magnitude dependent only on time. The waveform of the velocity magnitude can be characterized by the following three factors: maximum velocity value or peak systolic value (PSV); minimal velocity value or end diastolic value (EDV); and the time-averaged velocity (TAV) over the input plane. Since it was not possible to obtain patient-specific inlet velocity waveforms, the waveform was taken as an averaged curve over a set of patients such that the TAV over the input plane is fixed TAV = 0.3 m/s for all six cases. This resulted in different inlet velocity fluxes as the radii of inlet vessels were different in each case. The velocity and flux waveforms are shown in Figure 1, and the exact values of the flux and radii are collected in Table 2. For all six geometries, the heart rate was taken as 60 bpm.

**Table 2:** Inlet information: radius of the inlet artery (mm) and given inlet velocity (m/s).

Aneurysm designation	Radius (mm)	TAV	PSV	EDV	Inlet flux (mL/min)
A	2.089	0.3	0.45	0.2	247
B	2.194	0.3	0.45	0.2	272
C	1.909	0.3	0.45	0.2	206
D	2.153	0.3	0.45	0.2	262
E	1.924	0.3	0.45	0.2	209
F	1.593	0.3	0.45	0.2	143

PSV, peak systolic velocity; EDV, end diastolic velocity; TAV, time-averaged velocity. Inlet flux (mL/min) is computed from the TAV and the inlet area.

### 2.5 Hemodynamic quantities

The following hemodynamic quantities were considered based on Xiang et al. (3) and Axier et al. (18). Time-averaged wall shear stress (TAWSS) is defined as


$$\text{TAWSS}=\frac{1}{T}\int_{0}^{T}\left|WSS\right|dt,$$

where *T* denotes the time period of one cardiac cycle and WSS stands for wall shear stress vector. The oscillatory shear index (OSI) captures the directional changes of wall shear stress vector throughout one cardiac cycle. OSI is a non-dimensional quantity given by the relation


$$OSI=\frac{1}{2}\left(1-\frac{\left|\int_{0}^{T}WSS\;dt\right|}{\int_{0}^{T}\left|WSS\right|dt}\right)\text{.}$$

The low shear area (LSA) is the percentage ratio of the aneurysm area with the WSS less than 10% of the spatially averaged WSS and of the entire aneurysm area. Additionally, we focused on the following hemodynamic parameters: the peak wall shear stress (PWSS) and peak LSA—quantities evaluated at the time of the PSV, time-averaged LSA (TALSA)—integrated over one cardiac cycle, normalized TAWSS (NTAWSS), and normalized PWSS (NPWSS)—the percentage ratio between the TAWSS (resp. PWSS) over the dome and TAWSS (resp. PWSS) over the parent artery. All post-processing calculations were performed using Python and visualizations using ParaView (19).

### 2.6 *In vitro* models

**Figure 2:**
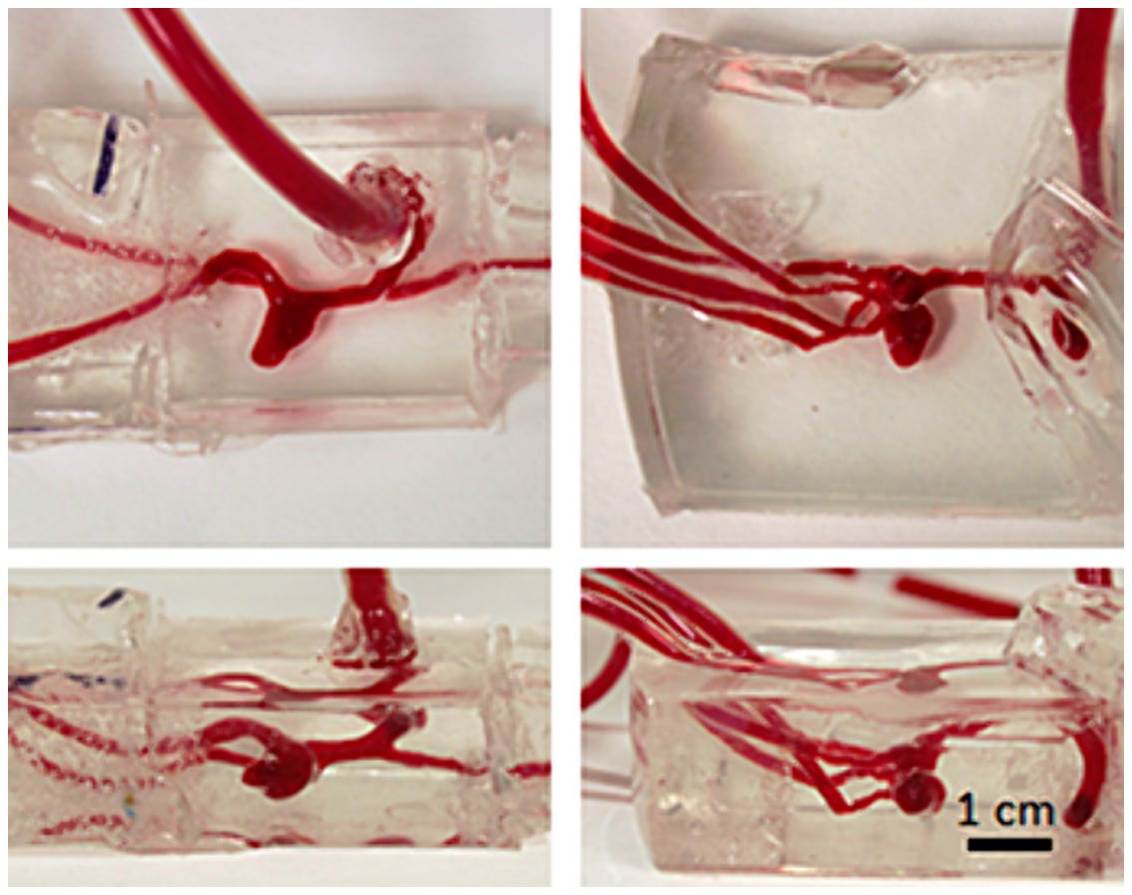
Silicone models: the lumen of the model was 3D printed out of water soluble material which was embedded into silicone together with inlet/outlet tubing and removed with hot water after silicone curing. The lumen of the final models was filled with phenol red solution to enhance visibility. Left column: model B, right column: model C.

The stereolithographic format (STL) of the aneurysm and connected arteries with patient-realistic dimensions was used to fabricate corresponding silicone models (Figure 2) using a 3D printer (Original Prusa i3MK2, Prusa Research, Czech Republic) and silicone (Sylgard 184, Dow Corning, United States). The lumen of the aneurysm and the corresponding arteries were 3D printed out of water-soluble material [butenediol vinyl alcohol (BVOH)]: verbatim BVOH filament soluble support (Verbatim/Mitsubishi Chemical Holdings Group, Japan). The printout with appropriate tubing was embedded in a silicone cast. The printout was then removed with distilled water. The dimensions of the model lumen were verified using an X-ray computed tomography (horizontal resolution 0.2 mm, vertical resolution 0.5 mm).

### 2.7 Particle velocimetry

Each model was placed in an open-flow loop with steady flow. The inlet flux was fixed at 169 mL/min. All flow parameters are displayed in Table 3.

**Table 3:** Flow parameters used for the comparison of CFD and PIV.

Model	radius (mm)	Inlet flux (mL/min)	TAV (m/s)
B	2.194	169	0.19
C	1.909	169	0.25

The inlet flux was fixed.

**Figure 3:**
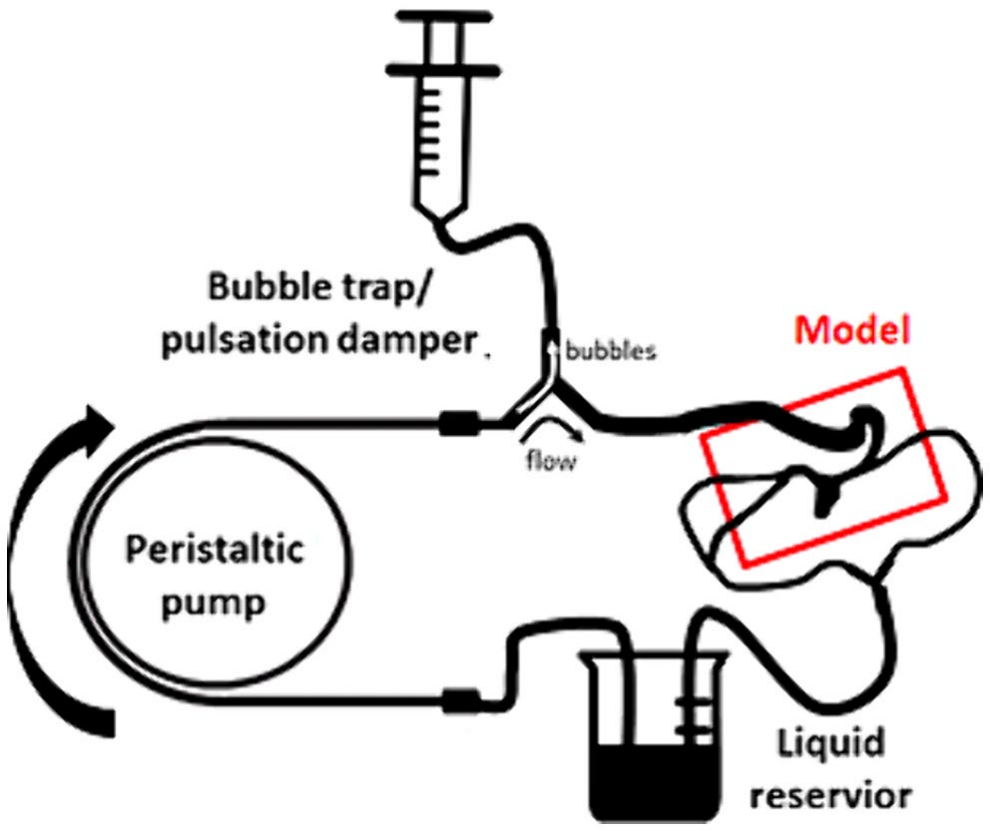
Scheme of the setup for the particle velocimetry.

The inlet of a peristaltic pump (Gilson Miniplus 3, high flow rate head, 8 mm ID tubing for peristaltic loop) was placed in a reservoir. A bubble trap and a pulsation damper were located behind the pump. The damper was made of a “Y” tube connector with a 60 mL syringe connected through a 50 cm long tube followed by the inlet into a silicone model. All model outlets were joined, and the common outlet was placed into the reservoir (Figure 3). The internal diameter of the tubing was kept larger than that of the model inlets/outlets. As a circulating medium served 47% (Vol/Vol) glycerin in water containing 0.1% (Vol/Vol) Tween 20 and supplemented with a suspension of 30 μm polystyrene beads (Cat No. 95531, Merck, Germany). The density of the circulating medium was 1,125 kg m^−3^. To maintain viscosity of 3.75 mPa s the flow loop was placed in a thermostatic chamber (38°C, Okolab, Italy) at Axio Observer Z1 microscope by Zeiss (Germany). The microscope was equipped with a 2.5× Apo Plan objective (NA 0.06) and Orca 4.0 LT digital camera (Hamamatsu, Japan). The focal depth of the setup was 248 μm. The flow velocity was evaluated by means of particle traces length within the exposure time (1–10 ms) using image analysis in Image J (20). The distance of the measurement plane from the base was determined by calibrated vertical movement of the microscope stage.

### 2.8 Comparison between CFD and PIV

Kinematic similarity between CFD simulations and PIV measurements is attained by maintaining the Reynolds number. In our case, it was achieved by setting consistent ratios between the fluid density and dynamic viscosity.

The measurement was performed in a plane parallel to the base of the model, the plane was set by the focus of the camera in the PIV experiment. The line segment was set at the beginning of the measurement so that one end of the line lied on the top of the aneurysm. The same plane and line segment in the plane were then found in ParaView using Geometric Shapes and CT scans of the printed models. Computed velocities were projected onto three different line segments with the same slope: one of them lay exactly in the measurement plane and the other two lines were shifted up and down by the distance of depth of field (DOF) of the microscope. Velocity projection was performed in Python using function *sample_over_line* from PyVista library (21). Moreover, traces of the massless particles were tracked using ParaView. We created randomly distributed particles in the aneurysm dome and tracked their path lines for 10 ms which corresponds to the exposition time of the camera.

## 3 Results

### 3.1 CFD modeling

Six patients with ruptured IAs were analyzed. All 6 patients were admitted for IA rupture and were operated on in an emergent setting (within 24 h after rupture). The point of rupture was identified during surgical exploration of the aneurysm before clipping and it was marked on the CTA 3D image after surgery. In one case (aneurysm A), a leak of blood from the aneurysm sac was clearly visible on preoperative CT angiography.

The results of the mesh sensitivity test are shown in Table 4. Steady-state quantities, namely the velocity, pressure and WSS, were averaged over the aneurysm dome using three meshes with different average edge lengths. Space-averaged velocity and pressure computed on three different geometries either remain constant or vary on the third significant digit. WSS values depend on mesh refinement in most cases. The finest meshes with an average edge length between 0.173 mm and 0.244 mm were then used for the evaluation of hemodynamics.

**Table 4:** Results of the mesh dependency test.

A				D			
*h* (mm)	*v* (m/s)	*p* (Pa)	WSS (Pa)	*h* (mm)	*v* (m/s)	*p* (Pa)	WSS (Pa)
0.239	0.277	1,649	1.425	0.306	0.182	207	0.233
0.220	0.277	1,634	1.427	0.279	0.179	204	0.219
0.192	0.277	1,611	1.434	0.244	0.179	204	0.215

B				E			
*h* (mm)	*v* (m/s)	*p* (Pa)	WSS (Pa)	*h* (mm)	*v* (m/s)	*p* (Pa)	WSS (Pa)
0.241	0.214	299	1.520	0.238	0.269	436	0.635
0.229	0.214	300	1.569	0.192	0.269	430	0.660
0.215	0.215	300	1.585	0.185	0.269	430	0.667

C				F			
*h* (mm)	*v* (m/s)	*p* (Pa)	WSS (Pa)	*h* (mm)	*v* (m/s)	*p* (Pa)	WSS (Pa)
0.221	0.295	681	0.477	0.241	0.285	267	0.940
0.214	0.295	680	0.479	0.184	0.286	264	1.001
0.191	0.296	678	0.476	0.173	0.286	263	1.015

An average edge length *h* in millimeters, velocity magnitude *v* in meters per second, normal pressure *p* and wall shear stress WSS in pascals. Values were obtained as the mean over the aneurysm area after the flow was steady.

The rupture sites were marked by operating neurosurgeons on 3D CT angiography reconstructions taken after rupture. These areas were determined as intersections of computational meshes with a sphere with a radius of 0.5 mm.

The resulting hemodynamic parameters, which can be found in Table 5, were averaged over the aneurysm dome. It can be seen that the PWSS values are nearly two times larger than TAWSS values. Both parameters were additionally normalized with respect to the corresponding parent artery. The PLSA and TALSA in cases A–E in comparison with case F were significantly smaller. However, due to averaging over the aneurysm dome, it is very difficult to distinguish between different hemodynamic cases solely from these values. Thus, we considered the spatial distribution of hemodynamic quantities in the subsequent analysis. We exploit the fact that the rupture site is known and evaluate the OSI and TAWSS averaged both over the dome and over the rupture site and their relative percentage differences, see Table 6. In models A–D there was moderate decrease of TAWSS at the ruptured site compared to the TAWSS over the respective domes, which was even more pronounced in model F. On the contrary, the opposite ratio, significantly higher TAWSS compared to the average TAWSS over the dome, was found in model E.

**Table 5:** Values of the hemodynamic parameters over the aneurysm dome.

Over the dome	A	B	C	D	E	F
PWSS aver (Pa)	5.199	5.196	1.492	1.144	1.932	1.380
PWSS max (Pa)	48.17	25.44	13.06	8.81	25.15	19.81
TAWSS aver (Pa)	2.764	3.145	0.776	0.683	1.056	0.834
TAWSS max (Pa)	27.75	12.85	6.82	4.06	14.26	11.24
OSI aver	0.0082	0.0866	0.0144	0.0242	0.0143	0.0129
OSI max	0.37	0.48	0.37	0.48	0.46	0.45
NTAWSS (%)	33.85	41.98	8.32	21.32	26.19	21.70
NPWSS (%)	43.26	44.63	9.82	21.47	29.93	21.18
PLSA (%)	0.46	0.34	0.17	1.84	2.91	16.45
TALSA (%)	0.00	0.00	0.00	0.0	0.25	14.24

Max denotes the maximum over the dome and aver denotes the average value over the dome. PWSS, peak wall shear stress; TAWSS, time-averaged wall shear stress; OSI, oscillatory shear index; NTAWSS, normalized time-averaged wall shear stress; NPWSS, normalized peak wall shear stress; PLSA, peak low shear area; TALSA, time-averaged low shear area.

**Table 6:** The OSI and TAWSS values were averaged over the dome and over the rupture site.

	A	B	C	D	E	F
TAWSS dome (Pa)	2.764	3.145	0.776	0.683	1.056	0.834
TAWSS rupture site (Pa)	1.022	2.728	0.2	0.265	2.861	0.017
relative difference (%)	−63.0	−13.3	−74.2	−61.2	170.9	−98.0
OSI dome	0.0082	0.0866	0.0144	0.0242	0.0143	0.0129
OSI rupture site	0.0163	0.1547	0.0526	0.0699	0.004	0.0063
Relative difference (%)	98.8	78.6	265.3	188.8	−72.0	−51.2

Relative percentage differences were obtained with respect to the average value over the dome.

**Figure 4:**
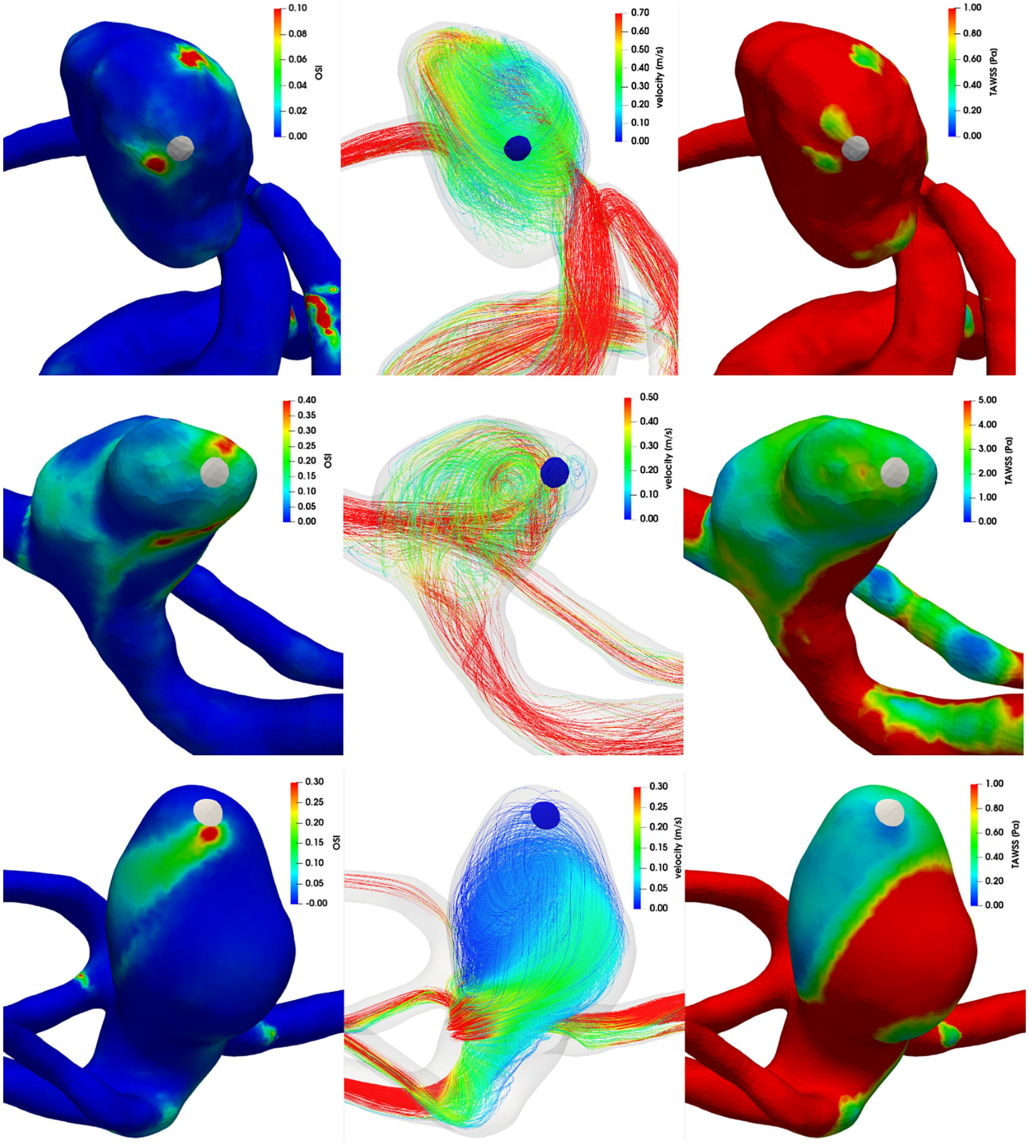
Left column: OSI, middle column: streamlines colored by velocity magnitude, right column: TAWSS. First row: aneurysm A, second row: aneurysm B, third row: aneurysm C.

**Figure 5:**
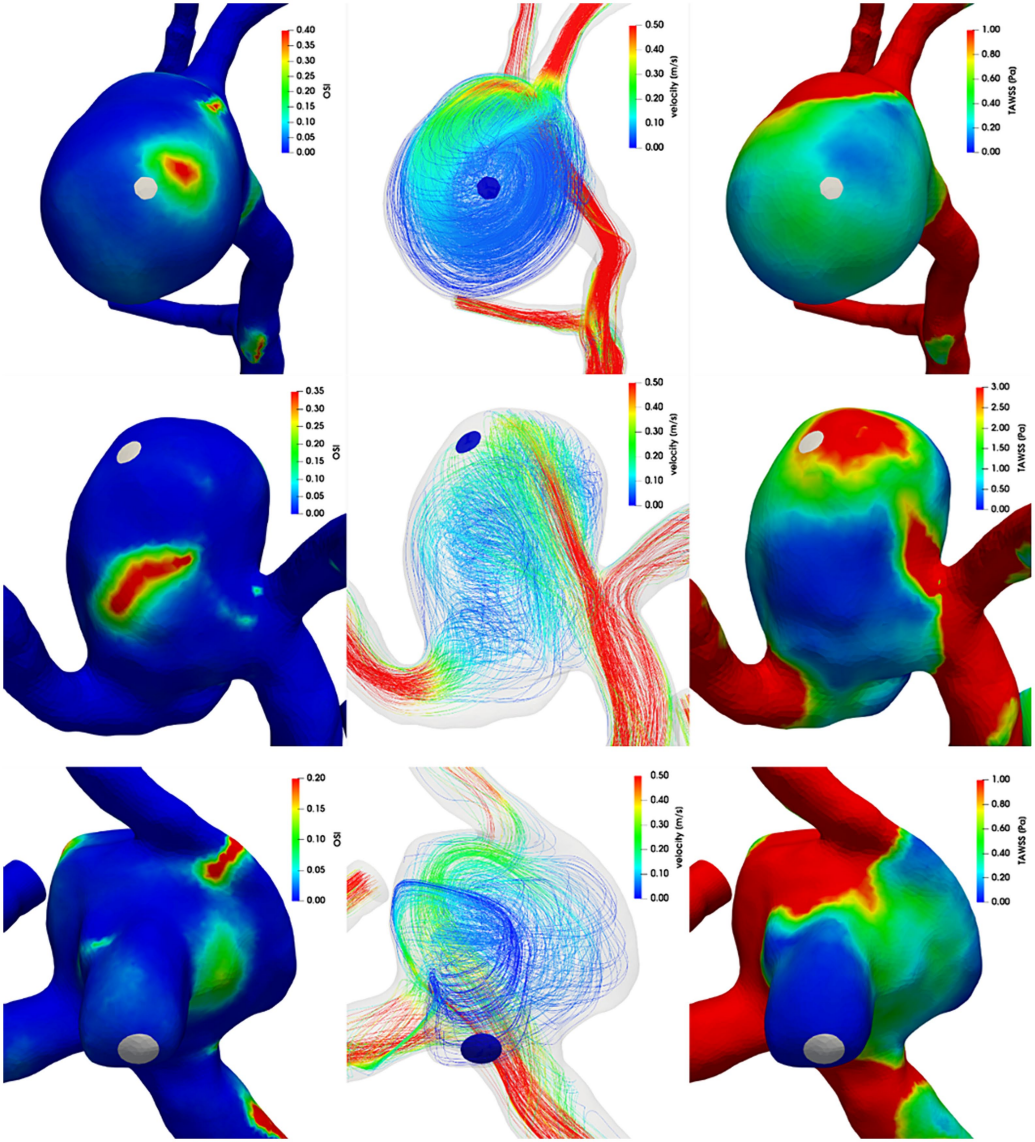
Left column: OSI, middle column: streamlines colored by velocity magnitude, right column: TAWSS. First row: aneurysm D, second row: aneurysm E, third row: aneurysm F.

Figures 4, 5 show the distribution of OSI and TAWSS in each aneurysm, as well as streamlines within the domain. Aneurysms A–D had ruptured near the vortex with high OSI, aneurysm E had ruptured near the flow jet with high WSS, and aneurysm F had ruptured on the bleb. In each figure, the rupture sites are colored white or blue.

### 3.2 CFD validation with particle velocimetry

**Figure 6:**
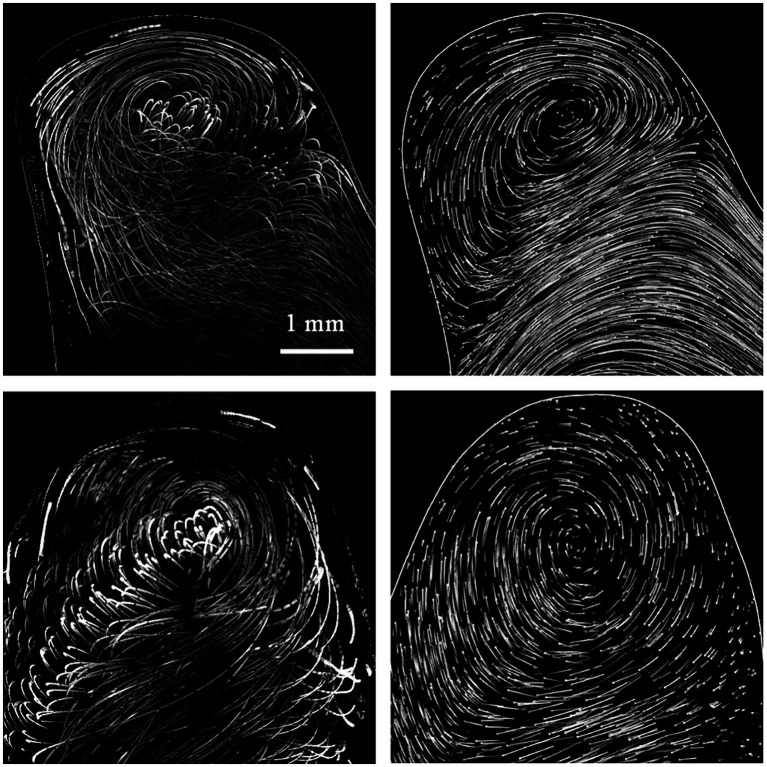
Left column: PIV, right column: CFD results, first row model A, second row model B. The result of PIV measurement shows sum of 10 consecutive images (focal depth 247 μm) taken at 97 Hz with 10 ms exposure with 30 μm polystyrene particle driven by flow. Thus, the traces were rather nonuniformly distributed and are laid in rows. The CFD shows the 10 ms traces of massless randomly distributed particles. The end of the trace is denoted by a dot. In order to simulate the PIV image with high fidelity the traces of particles within focal depth are displayed and coded with greyscale tones to denote the distance from the center plane of the focal depth.

**Figure 7:**
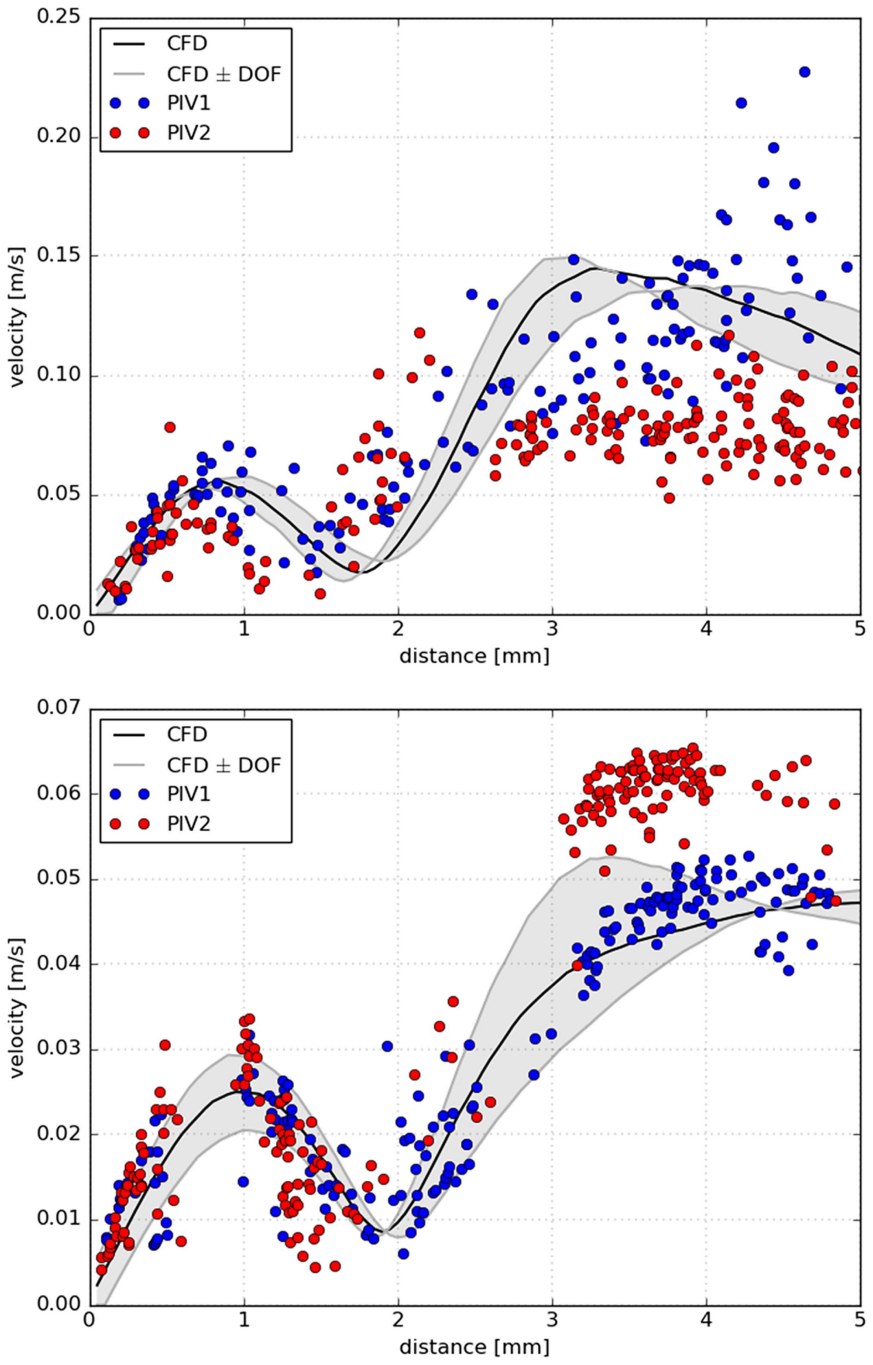
Measured velocities along the line segment obtained by two PIV measurements (blue and red circles) together with computed velocities via CFD projected to the same line. The line was projected from the top of the aneurysm dome where rupture occurred through the center of the aneurysm in the measurement plane (black line); upper panel: aneurysm B, lower panel: aneurysm C. In particular, the black line represents computed velocities in the measurement plane, while the grey lines denote velocities at the border of the focal depth (247 μm) of PIV. For clarity, the space between the border lines was highlighted with light gray color. In PIV measurement 1 (PIV1) the laboratory model position was aligned with CFD mesh precisely. Whereas in the 2nd PIV measurement (PIV2) the position of laboratory model was taken from PIV1.

Throughout CFD analysis there was some uncertainty about the prescribed boundary conditions and inlet flow profile. Therefore, validation using a laboratory model was carried out. We chose model B and C for this purpose. There was steady flow at a generic value of 169 mL/min with a parabolic profile and zero outlet pressure applied for both models. The same conditions were set for laboratory models (i.e., pulsation damper after peristaltic pump, volumetric control of the flow, open system with tubing diameter exceeding the diameters of the inlet or outlet of the laboratory model). Qualitative characteristics such as flow direction, position of flow jets and central vortexes in the aneurysm dome were very similar between CFD and PIV in the laboratory model shown in Figure 6. In order to provide thorough support to CFD data, quantitative analysis was carried out. Flow profiles were determined by PIV and computed by CFD within the focal depth of the PIV system. Concerning the precise alignment of laboratory models with CFD model meshes (PIV measurement 1 in Figure 7), good conformity was achieved between CFD and PIV data. Repeated positioning of the laboratory model under the microscope stage could result in imprecisions as seen in PIV measurement 2 in Figure 7. Therefore, matching individual PIV measurements could be worse than matching PIV to CFD, if inaccurate positioning of the model occurred. The PIV measurement was charged with a certain variance of data, see Figure 7. This could be attributed to the finite size (30 μm) and density (1,050 kg m^−3^) of particles compared to massless particles used in CFD.

## 4 Discussion

There has been a slow, but gradual increase of interest in hemodynamics of IAs in the field of clinical neurosurgery. The field of hemodynamics has been studied from different perspectives, such as their relationship to IA development, growth or rupture and some hemodynamic parameters have been integrated in newly developed risk factor scoring systems to help physicians estimate the risk of rupture (2, 3). In recent years, few studies have focused on local hemodynamic parameters associated with the site of rupture in IAs (5). Due to difficulties in identifying the precise point of rupture, the majority of such studies consisted of case reports or small mini-series.

These mini-series presented divergent hemodynamic scenarios. Interestingly, the site of rupture was characterized by a uniform hemodynamic environment within each study. We believe it is the first time that we described divergent hemodynamic scenarios at the site of rupture within a single study. Most aneurysms (*n* = 4) in this study, had a rupture site associated with an area of low WSS and increased OSI compared to the aneurysm dome as a whole. In another aneurysm, the site of rupture was associated with a jet flow directed against the aneurysm sac and very low OSI compared to the aneurysm dome as a whole. We have already described this type of hemodynamic environment in our former case report in a ruptured anterior communicating artery aneurysm (22). In the last case, the site of rupture was within a bleb of the aneurysm dome and was associated with low WSS and low OSI.

These typical hemodynamic scenarios have been described in previous studies (5). Our own study consisted of a relatively uniform series of aneurysms. Most of the IA, except for 1, were MCA aneurysms and all of them were large-size aneurysms, ranging from 8 to 13 mm in the largest diameter. Despite the relative homogeneity of aneurysm size and location, hemodynamics at the point of rupture differed significantly. This is of interest, because from a clinical perspective all of these aneurysms, if unruptured, would be viewed similarly when discussed in terms of rupture risk.

**Figure 8:**
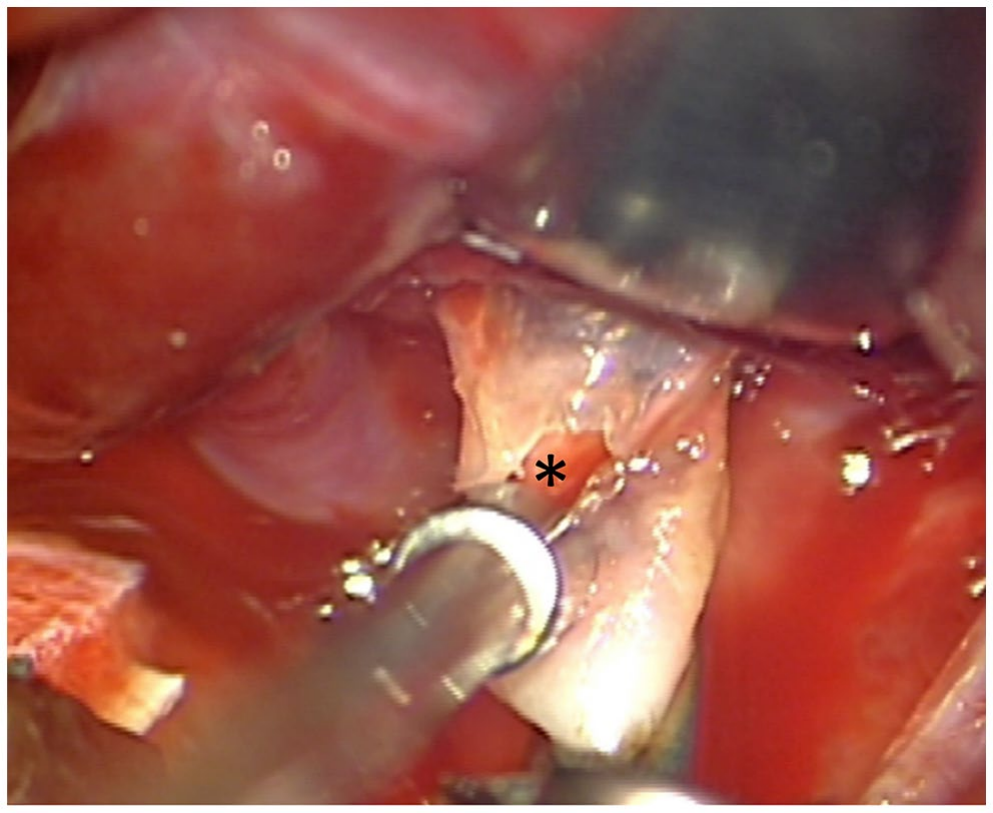
The point of rupture in aneurysm E (asterisk). The wall at the site of rupture is translucent. In this model the site of rupture was associated with high WSS compared to the rest of the dome.

Specific hemodynamic parameters have been shown to be related to different qualities of the aneurysm wall (23). In that study jet flow and high WSS were associated with thin, translucent regions of the aneurysm wall. Our perioperative photo of the only aneurysm whose point of rupture was defined by high WSS and jet flow demonstrates highly translucent aneurysm wall (Figure 8). We have already described this hemodynamic environment associated with aneurysm sac rupture.

On the other hand, most rupture points in our IAs showed low WSS together with high OSI. Such hemodynamics have been shown to be associated with thick, atherosclerotic regions. Atherosclerosis is often found in large aneurysms, which was the case of all the aneurysms in our study (24). Wall shear stress influences the morphology and orientation of endothelial cell within the vessel lumen. While high WSS may lead to their elongation and alignment, endothelial cells subjected to low WSS are rounded with no preferred alignment (25, 26). Moreover, exposure of the arterial wall to a relatively low wall shear stress may increase intercellular permeability and consequently increase the vulnerability of these regions of the vessel to atherosclerosis (5). Gradual enlargement of the LSA over time is associated with IA rupture (27).

One of the aneurysms in our series ruptured within the secondary sac or a bleb. Blebs seem to form at or near regions of high flow regions and elevated WSS (28). After bleb is formed it is characterized by low WSS as confirmed in this case. The high WSS associated with bleb development leads to thinning the aneurysm wall making it prone to rupture. In general, IA irregularities including secondary blebs are associated with an increased risk of aneurysm rupture (29–31).

Despite our study is based on a small series of IAs, it confirms the hypothesis that different pathophysiological pathways result in IA rupture (7). More specifically, while low WSS and high OSI may lead to activation of inflammatory cells (atherosclerosis), high WSS may lead to vessel wall cell activation resulting in inadequate growth and rupture (translucent aneurysm wall).

### 4.1 CFD model justification and validation with PIV

In CFD analysis we utilized the no-slip boundary condition on the vessel wall, zero pressure condition on each outlet part of the boundary, and parabolic profile of inlet flow velocity multiplied by a time-dependent waveform during a cardiac cycle. Concerning the proper choice of boundary conditions, there has been no general agreement on the optimal prescribed parameters. Recent studies found that both inflow and outflow boundary conditions have considerable impact on the hemodynamics (32). Commonly used inflow and outflow conditions are listed, for example (10). In this study, equal outlet pressures were the most frequently used outlet boundary conditions, while on the inlet, most groups prescribed a constant plug profile for either velocity or flow rate. However, in our case we had access to a set of velocity waveforms. Thus, the average time-dependent waveform is likely to model each patient-specific inlet boundary condition more realistically than a constant plug profile or flow rate.

To further address uncertainties about CFD analysis conditions and to evaluate the match of CFD to real flow, a validation using PIV in a laboratory model was carried out. For this purpose, two models (B and C, with the most complex inlet/outlet structures) were chosen. We capitalized on steady inflow with a parabolic profile in order to match the laboratory model with a pump equipped with pulsation damper and connected to tubing. This apparatus in principle develops a parabolic (laminar) flow profile at a flow speed up to several meters per second.

Under these conditions we observed a good qualitative agreement of CFD and PIV as the flow direction, position of flow jets and vortexes matched well. The quantitative agreement of CFD and PIV was well proven by determining flow profiles at line segments from the top of the aneurysm to the center of the aneurysm dome. If the laboratory model was aligned properly with the CFD model mesh there was agreement between CFD and PIV, which was similar or better than reported in literature (33–37). The possible limitation of this approach arose from imprecisions caused by repeated laboratory model positioning for PIV measurement.

### 4.2 Study limitations

Despite providing reasonable data the study has few limitations which should be taken into consideration. Since the primary focus was the relationship of aneurysm rupture and hemodynamics, the study did not consider possible co-morbidities (smoking, hypertension, dyslipidemia, etc.) which can affect the aneurysm development and rupture. Specific limitations of studies in ruptured aneurysms result from the fact that we already assess the condition given by rupture, not the aneurysm at risk, before rupture. Therefore, altered morphology of the aneurysm after rupture can lead to erroneous results or misinterpretations (38). Another limitation in these studies is that we usually do not possess the individual boundary conditions for each aneurysm and need to use literature-based information. In our CFD analysis, we employed a straightforward choice of boundary conditions. We prescribed a generic parabolic velocity profile at the inlet and applied a do-nothing condition on all outlet boundaries. Such an approach may introduce a bias into the CFD analysis if processing aneurysm model based on a patient with anomalous inlet/outlet boundaries.

## 5 Conclusion

Using a PIV validated CFD model of a ruptured aneurysm, we evaluated hemodynamics of 6 ruptured aneurysms. The most important finding was identification of rupture points associated with various hemodynamic features (low WSS with recirculations contrary to high WSS with a jet flow). Hence our data indicates that divergent biomechanical pathways are involved in the mechanism of aneurysm rupture.

## Data availability statement

The original contributions presented in the study are included in the article/supplementary material, further inquiries can be directed to the corresponding author.

## Ethics statement

The studies involving humans were approved by Ethics Committee of the Krajská zdravotní, a.s. The studies were conducted in accordance with the local legislation and institutional requirements. Written informed consent for participation was not required from the participants or the participants’ legal guardians/next of kin in accordance with the national legislation and institutional requirements.

## Author contributions

AH: Conceptualization, Data curation, Formal analysis, Funding acquisition, Investigation, Methodology, Project administration, Resources, Supervision, Writing – original draft, Writing – review & editing. JB: Formal analysis, Methodology, Writing – original draft, Writing – review & editing. HŠ: Conceptualization, Data curation, Formal analysis, Investigation, Methodology, Writing – original draft, Writing – review & editing. JV: Data curation, Formal analysis, Funding acquisition, Investigation, Methodology, Resources, Writing – original draft, Writing – review & editing. AW: Conceptualization, Data curation, Investigation, Methodology, Writing – review & editing. AS: Investigation, Methodology, Writing – review & editing. MSt: Data curation, Methodology, Writing – review & editing. TR: Data curation, Methodology, Writing – review & editing. MSa: Supervision, Writing – review & editing, Conceptualization. JH: Data curation, Investigation, Methodology, Project administration, Resources, Validation, Writing – review & editing.

## References

[1] Gabriel RA Kim H Sidney S McCulloch CE Singh V Johnston SC. Ten-year detection rate of brain arteriovenous malformations in a large, multiethnic, defined population. Stroke. (2010) 41:21–6. doi: 10.1161/STROKEAHA.109.566018, PMID: 19926839 PMC2847493

[2] Dhar S Tremmel M Mocco J Kim M Yamamoto J Siddiqui AH. Morphology parameters for intracranial aneurysm rupture risk assessment. Neurosurgery. (2008) 63:185–96. doi: 10.1227/01.NEU.0000316847.64140.81, PMID: 18797347 PMC2570753

[3] Xiang J Natarajan SK Tremmel M Ma D Mocco J Hopkins LN. Hemodynamic-morphologic discriminants for intracranial aneurysm rupture. Stroke. (2011) 42:144–52. doi: 10.1161/STROKEAHA.110.592923, PMID: 21106956 PMC3021316

[4] Xiang J Yu J Choi H Dolan Fox JM Snyder KV Levy EI. Rupture resemblance score (RRS): toward risk stratification of unruptured intracranial aneurysms using hemodynamic-morphological discriminants. J Neurointerv Surg. (2015) 7:490–5. doi: 10.1136/neurintsurg-2014-011218, PMID: 24811740 PMC6383516

[5] Jiang Y Lu G Ge L Huang L Wan H Wan J. Rupture point hemodynamics of intracranial aneurysms: case report and literature review. Ann Vasc Surg. (2021) 1:100022. doi: 10.1016/j.avsurg.2021.100022

[6] Zhang Y Jing L Zhang Y Liu J Yang X. Low wall shear stress is associated with the rupture of intracranial aneurysm with known rupture point: case report and literature review. BMC Neurol. (2016) 16:231. doi: 10.1186/s12883-016-0759-0, PMID: 27863464 PMC5116170

[7] Meng H Tutino VM Xiang J Siddiqui A. High WSS or low WSS? Complex interactions of hemodynamics with intracranial aneurysm initiation, growth, and rupture: toward a unifying hypothesis. AJNR Am J Neuroradiol. (2014) 35:1254–62. doi: 10.3174/ajnr.A3558, PMID: 23598838 PMC7966576

[8] Staarmann B Smith M Prestigiacomo CJ. Shear stress and aneurysms: a review. Neurosurg Focus. (2019) 47:E2. doi: 10.3171/2019.4.FOCUS19225 31261124

[9] Ujiie H Tamano Y Sasaki K Hori T. Is the aspect ratio a reliable index for predicting the rupture of a saccular aneurysm? Neurosurgery. (2001) 48:495–502. doi: 10.1097/00006123-200103000-00007, PMID: 11270538

[10] Berg P Roloff C Beuing O Voss S Sugiyama S-I Aristokleous N. The computational fluid dynamics rupture challenge 2013—phase II: variability of hemodynamic simulations in two intracranial aneurysms. J Biomech Eng. (2015) 137:121008. doi: 10.1115/1.4031794, PMID: 26473395

[11] Yushkevich PA Piven J Hazlett HC Smith RG Ho S Gee JC. User-guided 3D active contour segmentation of anatomical structures: significantly improved efficiency and reliability. NeuroImage. (2006) 31:1116–28. doi: 10.1016/j.neuroimage.2006.01.015, PMID: 16545965

[12] Fang Q Boas DA (2009). Tetrahedral mesh generation from volumetric binary and grayscale images. 2009 IEEE International Symposium on Biomedical Imaging: From Nano to Macro. Boston, MA: IEEE, 1142–1145

[13] Magjarevic R Poethke J Spuler A Petz C Hege H-C Goubergrits L. (2009). Cerebral aneurysm hemodynamics and a length of parent vessel. Dössel O. Schlegel W. C. World Congress on Medical Physics and Biomedical Engineering. September 7–12, 2009. Munich, Germany. (Berlin: Springer), 1608–1611

[14] Chabiniok R Hron J Jarolímová A Málek J Rajagopal KR Rajagopal K. Three-dimensional flows of incompressible Navier–Stokes fluids in tubes containing a sinus, with varying slip conditions at the wall. Int J Eng Sci. (2022) 180:103749. doi: 10.1016/j.ijengsci.2022.103749

[15] Alnæs M Blechta J Hake J Johansson A Kehlet B Logg A. The FEniCS project version 1.5. Arch Numer Softw. (2015) 3:9–23. doi: 10.11588/ANS.2015.100.20553

[16] Abhyankar S Brown J Constantinescu EM Ghosh D Smith BF Zhang H (2018). PETSc/TS: a modern scalable ODE/DAE solver library. *arXiv*. Available at: http://arxiv.org/abs/1806.01437 (Accessed July 19, 2022) [Epub ahead of preprint].

[17] Arnold DN Brezzi F Fortin M. A stable finite element for the stokes equations. Calcolo. (1984) 21:337–44. doi: 10.1007/BF02576171

[18] Axier A Rexiati N Wang Z Cheng X Su R Aikeremu R. Effect of hemodynamic changes on the risk of intracranial aneurysm rupture: a systematic review and meta-analysis. Am J Transl Res. (2022) 14:4638–47. PMID: 35958447 PMC9360874

[19] Ayachit U. The ParaView Guide: A Parallel Visualization Application. Clifton Park, NY, USA: Kitware, Inc. (2015).

[20] Schneider CA Rasband WS Eliceiri KW Schindelin J Arganda-Carreras I Frise E. NIH image to ImageJ: 25 years of image analysis. Nat Methods. (2012) 9:671–5. doi: 10.1038/nmeth.2089, PMID: 22930834 PMC5554542

[21] Sullivan C Kaszynski A. PyVista: 3D plotting and mesh analysis through a streamlined interface for the visualization toolkit (VTK). J Open Source Softw. (2019) 4:1450. doi: 10.21105/joss.01450

[22] Hejčl A Švihlová H Sejkorová A Radovnický T Adámek D Hron J. Computational fluid dynamics of a fatal ruptured anterior communicating artery aneurysm. J Neurol Surg A. (2017) 78:610–6. doi: 10.1055/s-0037-1604286, PMID: 28800663

[23] Cebral JR Detmer F Chung BJ Choque-Velasquez J Rezai B Lehto H. Local hemodynamic conditions associated with focal changes in the intracranial aneurysm wall. AJNR Am J Neuroradiol. (2019) 40:510–6. doi: 10.3174/ajnr.A5970, PMID: 30733253 PMC6420361

[24] Kosierkiewicz TA Factor SM Dickson DW. Immunocytochemical studies of atherosclerotic lesions of cerebral berry aneurysms. J Neuropathol Exp Neurol. (1994) 53:399–406. doi: 10.1097/00005072-199407000-00012, PMID: 8021714

[25] Levesque MJ Liepsch D Moravec S Nerem RM. Correlation of endothelial cell shape and wall shear stress in a stenosed dog aorta. Arteriosclerosis. (1986) 6:220–9. doi: 10.1161/01.ATV.6.2.220, PMID: 3954676

[26] Levesque MJ Nerem RM. The elongation and orientation of cultured endothelial cells in response to shear stress. J Biomech Eng. (1985) 107:341–7. doi: 10.1115/1.3138567 4079361

[27] Sejkorová A Švihlová H Ondra P Kendall DD Uthamaraj S Lanzino G. Hemodynamic changes in four aneurysms leading to their rupture at follow-up periods. Ces Slov Neurol Neurochir. (2020) 83:621–6. doi: 10.48095/cccsnn2020621

[28] Cebral JR Sheridan M Putman CM. Hemodynamics and bleb formation in intracranial aneurysms. AJNR Am J Neuroradiol. (2010) 31:304–10. doi: 10.3174/ajnr.A1819, PMID: 19797790 PMC2859623

[29] Beck J Rohde S El Beltagy M Zimmermann M Berkefeld J Seifert V. Difference in configuration of ruptured and unruptured intracranial aneurysms determined by biplanar digital subtraction angiography. Acta Neurochir. (2003) 145:861–5. doi: 10.1007/s00701-003-0124-0, PMID: 14577007

[30] Suga M Yamamoto Y Sunami N Abe T Kondo A. Growth of asymptomatic unruptured aneurysms in follow-up study: report of three cases. No Shinkei Geka. (2003) 31:303–8. PMID: 12684985

[31] Tsukahara T Murakami N Sakurai Y Yonekura M Takahashi T Inoue T. Treatment of unruptured cerebral aneurysms; a multi-center study at Japanese national hospitals. Acta Neurochir Suppl. (2005) 94:77–85. doi: 10.1007/3-211-27911-3_12 16060244

[32] Li B Liu T Liu J Liu Y Cao B Zhao X. Reliability of using generic flow conditions to quantify aneurysmal haemodynamics: a comparison against simulations incorporating boundary conditions measured *in vivo*. Comput Methods Prog Biomed. (2022) 225:107034. doi: 10.1016/j.cmpb.2022.107034, PMID: 35914441

[33] Bordás R Seshadhri S Janiga G Skalej M Thévenin D. Experimental validation of numerical simulations on a cerebral aneurysm phantom model. Interv Med Appl Sci. (2012) 4:193–205. doi: 10.1556/imas.4.2012.4.4, PMID: 24265876 PMC3831782

[34] Brindise MC Rothenberger S Dickerhoff B Schnell S Markl M Saloner D. Multi-modality cerebral aneurysm haemodynamic analysis: *in vivo* 4D flow MRI, *in vitro* volumetric particle velocimetry and *in silico* computational fluid dynamics. J R Soc Interface. (2019) 16:20190465. doi: 10.1098/rsif.2019.0465, PMID: 31506043 PMC6769317

[35] Buchmann NA Yamamoto M Jermy M David T. Particle image velocimetry (PIV) and computational fluid dynamics (CFD) modelling of carotid artery haemodynamics under steady flow: a validation study. J Biomech Sci Eng. (2010) 5:421–36. doi: 10.1299/jbse.5.421

[36] Li Y Verrelli DI Yang W Qian Y Chong W. A pilot validation of CFD model results against PIV observations of haemodynamics in intracranial aneurysms treated with flow-diverting stents. J Biomech. (2020) 100:109590. doi: 10.1016/j.jbiomech.2019.109590, PMID: 31902608

[37] Roloff C Stucht D Beuing O Berg P. Comparison of intracranial aneurysm flow quantification techniques: standard PIV vs stereoscopic PIV vs tomographic PIV vs phase-contrast MRI vs CFD. J Neurointerv Surg. (2019) 11:275–82. doi: 10.1136/neurintsurg-2018-013921, PMID: 30061369

[38] Schneiders JJ Marquering HA van den Berg R VanBavel E Velthuis B Rinkel GJE. Rupture-associated changes of cerebral aneurysm geometry: high-resolution 3D imaging before and after rupture. AJNR Am J Neuroradiol. (2014) 35:1358–62. doi: 10.3174/ajnr.A3866, PMID: 24557706 PMC7966588

